# Critical Review on Toxicological Mechanisms Triggered by Inhalation of Alumina Nanoparticles on to the Lungs

**DOI:** 10.3390/biomedicines10102664

**Published:** 2022-10-21

**Authors:** Samir Dekali, Alexandra Bourgois, Sabine François

**Affiliations:** 1 French Armed Forces Biomedical Research Institute (IRBA), Department of Biological Radiation Effects/Unit of Emerging Technological Risks, 1 Place du Général Valérie André, BP 73, CEDEX, 91223 Brétigny-sur-Orge, France; 2 French Armed Forces Biomedical Research Institute (IRBA), Department of Biological Radiation Effects/Radiobiology Unit, 1 Place du Général Valérie André, BP 73, CEDEX, 91223 Brétigny-sur-Orge, France

**Keywords:** alumina nanoparticles, inflammation, fibrosis, toxicity, lung, broncho-alveolar fluid, aluminosis

## Abstract

Alumina nanoparticles (Al_2_O_3_ NPs) can be released in occupational environments in different contexts such as industry, defense, and aerospace. Workers can be exposed by inhalation to these NPs, for instance, through welding fumes or aerosolized propellant combustion residues. Several clinical and epidemiological studies have reported that inhalation of Al_2_O_3_ NPs could trigger aluminosis, inflammation in the lung parenchyma, respiratory symptoms such as cough or shortness of breath, and probably long-term pulmonary fibrosis. The present review is a critical update of the current knowledge on underlying toxicological, molecular, and cellular mechanisms induced by exposure to Al_2_O_3_ NPs in the lungs. A major part of animal studies also points out inflammatory cells and secreted biomarkers in broncho-alveolar lavage fluid (BALF) and blood serum, while in vitro studies on lung cells indicate contradictory results regarding the toxicity of these NPs.

## 1. Introduction

Alumina nanoparticles (Al_2_O_3_ NPs) are among the most widely used and produced particles in the world, with a wide range of interesting applications such as biosensors, desalination, high-risk pollutants detection, capacitors, solar cell devices, and photonic crystals [1]. These NPs can also be retrieved as pollutants in the environment.

Al_2_O_3_ NPs are produced in huge quantities as by-products of water treatment (water treatment residuals), bauxite processing (red mud), and hard and brown coal burning in power plants (fly ash) [2]. Welding fumes and propellant combustion residues also seem to be the main sources of Al_2_O_3_ NPs occupational exposures [3,4]. Nanoparticle forms are particularly studied in toxicological research because of their increasing use and the concerns they raise. Indeed, unique physico-chemical properties of NPs such as small size (<100 nm) and high specific surface area, confer high surface reactivity and uncertainty toward their potential toxicity [5]. Workers’ exposure to Al_2_O_3_ NPs can mainly occur by inhalation. Occupational exposures to these dusts are described in the literature to have deleterious health effects on the respiratory and nervous systems [3,6,7,8,9]. Therefore, it is necessary to improve understanding of NPs toxicity in order to redesign strategies to mitigate/reduce environmental and/or health impact [10]. Due to the lack of studies, research efforts are needed to better explore and thus protect predisposed populations such as workers.

This review focuses on biological effects described on the pulmonary system and associated described pathologies. Clinical, animal, and in vitro studies are successively presented in this paper. Briefly, workers exposed to Al_2_O_3_ NPs developed pneumoconiosis, also named “aluminosis”. They also presented local respiratory symptoms such as cough or shortness of breath, inflammation in the lung parenchyma, probably long-term pulmonary fibrosis, and increased risk of developing lung cancer [3,8,9,11]. Pulmonary aluminosis is a rare form of pneumoconiosis caused by aluminum or alumina powders [12,13]. Consequently, the majority of animal studies have focused on pro-inflammatory mechanisms triggered by the inhalation of Al_2_O_3_ NPs [14,15]. Results showed increases in total protein, neutrophils, lymphocytes, and lactate dehydrogenase (LDH) concentration in broncho-alveolar lavages (BALF), corroborating pro-inflammatory effects of Al_2_O_3_ NPs and suggesting potential permeabilization of the alveolo-capillary barrier. Moreover, various pro-inflammatory cytokines were secreted in animal BALFs: TNF-α, IL-6, IL-1β, IL-8, MIP-2, and IL-33 [14,15,16]. However, to the best of our knowledge, inflammatory mechanisms are poorly studied in vitro. This review also critically presents contradictory results on the cytotoxic and genotoxic effects of Al_2_O_3_ NPs on lung cells.

## 2. Clinical Studies on Health Effects after Inhalation of Alumina Particles

A recent study was conducted on fifteen male students aged (mean, range) 24, 19–31 with normal lung function in an inhalation chamber for human exposure to atmospheric particulate matter (PM) [9]. The aim was to examine the effects of short-term exposure to Al_2_O_3_ particles (3.2 μm, crystallinity unknown) on inflammatory markers in induced sputum in healthy volunteers. Controlled inhalations with exposure levels commonly seen in primary aluminum production were used (below toxicological reference values, i.e., 2 h at 3.9 mg/m^3^). Authors showed 24 h after exposure increases in polymorphonuclear neutrophils (PNNs), total proteins, and IL-8 concentrations in the sputum, suggesting a marked pulmonary inflammation. Moreover, microarray analyses of mRNA abundance collected from sputum macrophages and pathway analysis showed changes in the expression of 46 genes identified in three major biological process groups: cell–cell signaling, gene expression, RNA damage and repair, and regulation of connective tissue assembly. Only localized respiratory effects were reported in this study, and no systemic effects were observed.

A prevalence of pulmonary fibrosis 300 times higher than that observed in the general population was evaluated in workers exposed to alumina for 25 years [17]. However, these results are questionable because of the exposure of subjects to other particulate compounds that may have contributed to the onset of these respiratory pathologies. Indeed, significant quantities of asbestos fibers have also been found in the lungs of some subjects who have developed pulmonary fibrosis. Workers’ exposures to fumes from aluminum welding, which may contain alumina, has also led to the appearance of pneumoconiosis and pulmonary fibrosis in some cases [3,11]. A higher frequency of respiratory diseases, such as chronic obstructive pulmonary disease (COPD), has been observed in aluminum welders. In addition, decreases in lung function (forced expiratory volume in a second (FEV1)) have been observed in workers in the aluminum industry exposed to dust containing mainly alumina [18]. Another study evaluated the lung tissue of fourteen workers exposed to hard metals and aluminum oxide [19]. Among them, five workers underwent transbronchial biopsy showing diffuse interstitial inflammatory changes: two of them were asymptomatic, one had clinically evident disease with severe giant cell inflammation, and two other workers showed local inflammation.

Although some studies on alumina exposure have concluded the induction of respiratory pathologies and a decrease in lung function in humans, other studies have revealed contradictory results. Despite respiratory symptoms (cough, shortness of breath) observed in workers (preparers of powders for propulsion systems using duralium) exposed to aluminum and alumina NPs, no link between these compounds and the appearance of diseases respiratory problems could be demonstrated [8]. Similarly, the follow-up of a cohort of 521 men working in the production of abrasives (exposure to alumina particles, silicon carbides, and formaldehyde) 72 over 25 years did not show a significant increase in total or cancer mortality or the incidence of non-malignant respiratory disease [20]. Finally, monitoring of the pulmonary function of welders using aluminum did not show any deleterious effect, although respiratory symptoms were observed in these workers [21].

A summary of the results of clinical studies performed on health effects after inhalation exposure to alumina particles is available in Table 1.

## 3. Animal Studies of Pulmonary Biological Effects Triggered by Alumina Nanoparticles

In order to study the pulmonary toxicity of particles, different animal exposure methods can be implemented. Inhalation exposure is the most physiological method. However, this is expensive, and it requires extensive technical skills to set up the exposure system, the reproducibility of aerosol generation, and to characterize the physico-chemistry of generated aerosols. Another commonly used method to study in vivo lung toxicity of particles is intratracheal instillation (IT). This technique allows administrating of precise and known doses of particle suspensions [22]. IT is less constrained than inhalation but not representative of environmental exposure conditions. It is also possible to use other methods of exposure, such as nasal instillation, IT aspiration, oropharyngeal aspiration, or oral exposure. These techniques are less physiological and rarely used in literature.

Although possessing important anatomical and physiological similarities with humans, large mammals such as monkeys and pigs are very rarely used for lung toxicity studies due to ethical aspects and their cost. The rat is the alternative recommended by Organisation for Economic Co-operation and Development (OECD) because it is a more qualified model, easier to implement, and remains representative of the human respiratory system [23,24,25]. This animal model is preferred to the mouse model because it has more anatomical similarities with humans than with mice [26]. This animal model is the most commonly used to study the pulmonary toxicity of alumina particles. These studies are presented considering the kind of animal exposure, i.e., inhalation or IT/intranasal exposures.

### 3.1. Nose-Only and Whole-Body Inhalation Exposures

Kim and colleagues reported pro-inflammatory effects of alumina nanoparticles after repeated nose-only inhalation on Sprague-Dawley rats [15]. After 28 days of exposure (5 days/week) to Al_2_O_3_ NPs concentrations ranged between 0.2 and 5 mg/m^3^ (size 11.94 nm; unknown crystallinity), they showed increases in the total number of cells, neutrophils, lymphocytes, LDH, TNF-α, IL-6 in BALF. Moreover, they reported histopathological lesions with alveolar macrophage accumulation in four and eight cases of the 5 mg/m^3^ group during exposure and recovery, respectively. Pro-inflammatory effects significantly decreased after 28 days of exposure, but neutrophils and LDH concentrations remained significantly elevated compared to control groups. Results obtained in this study demonstrated a strong inflammatory potential of these NPs when inhaled, and authors suggested a no-observed-adverse-effect level of 1 mg/m^3^ concentration. Recently, Wistar rats were also nose-only exposed to a high concentration of Al_2_O_3_ NPs (size 13 nm; γ/δ crystallinity; 20.0–22.1 mg/m^3^ aerosol) following two exposure scenarios: single (4 h) or repeated exposures (4 h/day for 4 days) [14]. After repeated exposures, total proteins and LDH concentrations in BALF were significantly increased, suggesting that the alveolo-capillary barrier was damaged. Additionally, a marked pro-inflammatory reaction was observed with increased concentrations of neutrophils, macrophages, IL-1β, TNF-α, GRO/KC, and MIP-2. Moreover, another study showed that after 7 days of inhalation exposure of mice to Al_2_O_3_ NPs (size 40 nm; unknown crystallinity; dose of 0.4 mg/m^3^) in whole-body chamber emphysema and small airway remodeling in lungs can occur, accompanied by enhanced inflammation and apoptosis [16]. Authors demonstrated that protein tyrosine phosphatase, non-receptor type 6 (PTPN6), was down-regulated and Signal Transducer and Activator of Transcription 3 (STAT3) phosphorylated in response to Al_2_O_3_ NPs exposure, culminating in increased expression of the apoptotic marker Programmed cell death protein 4 (PDCD4). Moreover, IL-6 and IL-33 concentrations were significantly increased in BALF. Therefore, a decrease in PTPN6 may have deleterious effects at the molecular, cellular, and tissue levels, leading to the initiation of inflammation and apoptosis, ultimately resulting in the development of COPD-like lesions.

### 3.2. Intratracheal or Intranasal Exposures

IT or nasal instillation are the best alternatives to inhalation exposures for pulmonary toxicity studies in animals. These techniques notably offer the possibility of exposing animals in a less costly way, with good control of the dose administered in bolus and technically less complex than exposure by inhalation. In Sprague-Dawley rats exposed by IT to 40 mg of Al_2_O_3_ particles (size 4.37 μm, γ/α crystallinity), an increase in the number of cells, mainly macrophages but also neutrophils, and fibronectin concentrations were shown in BALF [27]. These concentrations remain increased twelve months after instillation, suggesting a persistence of the inflammatory phenomenon induced by Al_2_O_3_ NPs. These results are consistent with those obtained by inhalation but provide information on the persistence over time of the observed acute effects. BALF analysis of Wistar rats exposed to Al_2_O_3_ NPs (6.3 nm, crystallinity unknown, 0.5 mL at 300 cm^2^/mL) by IT also induced acute pulmonary inflammation [7]. Increases in polymorphonuclear cells were measured in BALF 24 h after exposure. In addition, Al_2_O_3_ NPs showed hemolytic potential. The effects observed in this study seem to be correlated with the surface properties of Al_2_O_3_ NPs (surface charge represented by the zeta potential in particular). Indeed, a correlation between the zeta potential and the influx of granulocytes or the hemolytic power has been observed for NPs with a high zeta potential [7].

Exposure by nasal instillation of Sprague-Dawley rats demonstrated other deleterious effects of alumina NPs (size and crystallinity unknown, 1–40 mg/kg) [28]. A slight dose-dependent inflammation was observed, but impairment of alveolo-capillary barrier permeability was also revealed by the increase in total protein concentration in BALF. The fibrotic potential of Al_2_O_3_ particles was assessed in Sprague-Dawley rats by IT and in NMRI mice by intraperitoneal injection [29]. The effects of different particles (different microparticle sizes; variable crystal polymorphs α, γ, δ, and χ) were evaluated. The study concluded that the particles usually used for the manufacture of aluminum (α and γ) had no fibrotic effect, whereas other particles tested could induce this type of lesion. Moreover, this study did not show any link between cytotoxic and fibrotic effects. Potential cardiac effects of Al_2_O_3_ NPs (size 11 nm; α crystallinity; 30 mg/kg/day; over 14 days) were studied on Sprague-Dawley rats after IT [30]. This study revealed adverse effects resulting in electrocardiogram (ECG) disorders and an increase in myocardial (LDH, triglycerides, creatine phosphokinase, cholesterol, nitric oxide) and inflammatory (TNF-α) damage markers. A decrease in antioxidants was also measured (reduced glutathione and superoxide dismutase) in animal serum.

A summary of the results of in vivo studies after inhalation/instillation exposure to alumina particles is available in Table 2.

## 4. In Vitro Studies of Cytotoxic Mechanisms Induced by Alumina Nanoparticles Exposure on Lung Cells

A study conducted on murine fibroblasts (L929 cell line) and normal human skin fibroblasts (BJ cells) incubated with 10 to 400 µg/mL of γ-Al_2_O_3_ particles (NPs fraction of 56–91 nm and agglomerates fraction of 106–220 nm) for 24 h did not show a decrease in cell viability nor apoptosis induction. However, the authors demonstrated the same penetration dynamics of Al_2_O_3_ particles in both cell types [31]. These results were confirmed by more recent experiments on human alveolar A549 epithelial cells and skin keratinocytes HaCaT exposed for 24 h to three different Al_2_O_3_ particles (primary sizes 14 nm, 111 nm, and 750 nm; α- and α/δ crystallinities; concentrations ranging from 10 to 50 mg/L) [32]. Particles were internalized by cells in the cytoplasm but not detected in nuclei and did not exert toxicity. A comparison of the cytotoxicity induced by different metal oxide NPs (Al_2_O_3_, CeO_2_, TiO_2_, and ZnO) on human lung cell lines (A549 carcinoma cells and L-132 normal cells) concluded lower cytotoxicity of NPs of alumina compared to the other NPs tested [33]. After 72 h incubation with Al_2_O_3_ NPs, no modification of the proliferation and cell viability were observed (size 20 nm, crystallinity unknown, NPs concentrations ranging from 1 to 1000 μg/mL). Moreover, the authors did not report a significant increase in LDH nor reactive oxygen species (ROS) production. Park and colleagues studied the toxicity of three types of synthesized aluminum oxide nanoparticles (AlONPs): γ-aluminum oxide hydroxide nanoparticles (γ-AlOHNPs), γ- and α-AlONPs (diameter 180–200 nm, exposure concentrations 5 and 20 μg/mL) [34]. They exposed for 24 h six human cell lines to NPs, including bronchial epithelial BEAS-2B cells, and showed that γ-AlOHNPs induced the greatest toxicity by decreasing ATP production and normalized cell index (ICN: parameter taking into account cell number, morphology, and cell adhesion), and increasing LDH release. They postulated that low stability in biological and hydroxyl groups of γ-AlOHNPs plays an important role in their cytotoxicity and bioaccumulation. Conflicting results are available in the literature regarding the effects of alumina particle exposure on cell proliferation. A decrease in ICN of bronchial epithelial cells (cell line 169HBE14o-) was shown after exposure to Al_2_O_3_ NPs (size less than 50 nm, crystallinity unknown) for 48 h [35]. Conversely, the exposure of pleural cells (NCI-H460 cell line) at similar concentrations of alumina NPs (14 nm, unknown crystallinity) did not show any significant modification of the ICN [36]. 

These studies seem to show that Al_2_O_3_ particles’ physico-chemical parameters (chemistry, size, etc.) and the cellular model used to determine the observed cytotoxicity. Particle size also seems to play the main role in cytotoxicity mechanisms. Indeed, human A549 alveolar epithelial cells were respectively exposed to Al_2_O_3_ NPs (sizes 10 nm and 50 nm; γ and γ/δ crystallinities) and titanium dioxide particles (TiO_2_, 5 nm, and 200 nm) for two and five days. Cell metabolism and cell proliferation were then studied using Alamarblue^®^ and clonogenic assays. Contrary to Kim et al., they showed that Al_2_O_3_ NPs were more cytotoxic than TiO_2_ [33,37]. Smaller NPs (according to their primary size) exhibiting higher relative surface area than larger particles also induced more toxic effects, but this was not correlated with measured hydrodynamic particle sizes (diameter of the NPs and/or agglomerates in a biological medium). This suggests that the more important the specific surface area of NPs is, the more Al_2_O_3_ NPs can be cytotoxic. However, the cytotoxicity of Al_2_O_3_ NPs may not only depend on particle size. Indeed, a study investigated genotoxic effects on human fibroblasts of different particles containing alumina (NPs, microparticles, and fibers) and did not put evidence of differences in the induction of micronuclei [38]. Authors hypothesized that biological effects after exposure to particles would appear to depend on chemical composition as well as size, shape, and cell type. Recently, Bourgois et al. also exposed 24 h A549 alveolar epithelial cells to Al_2_O_3_ particles [39]. They did not show any effects of different particle sizes and crystallinities on normalized cell index, cell viability, reduced glutathione, and double DNA strand breaks.

Although results of studies on Al_2_O_3_ NPs cytotoxicity do not seem to show systematically significant decreases in cell viability, these particles can induce different biological effects. It was shown that Al_2_O_3_ NPs (sizes 10–20 nm, crystallinity γ/α, concentrations ranging from 1 to 250 μg/mL) exposure for 24 h led to increases in mRNA and protein expression of VCAM-1, ICAM-1, and ELAM-1 in endothelial cells and increased adhesion of activated monocytes [40]. This study suggests the pro-inflammatory effects of alumina in nanoparticle form. Recently, experiments were performed on human bronchial epithelial (HBE) cells in order to characterize microRNA expression using microarrays after Al_2_O_3_ NPs exposure for 24 h (size distribution between 5 and 100 nm, unknown crystallinity, concentrations of 50 and 250 μg/mL) [41]. A homologous miRNA in Homo sapiens and Mus musculus, miR-297, was significantly up-regulated following exposures to Al_2_O_3_ NPs compared to control cells. Moreover, a few studies have reported a genotoxic potential of alumina NPs in vitro. An increased frequency of micronuclei and chromosomal aberrations has been observed on primary cultures of human fibroblasts exposed to alumina NPs (size 0.2 μm, crystallinity unknown, concentration ranging from 0.1 to 10 mg/culture flask). Nevertheless, the genotoxic effects induced were less important than those obtained in parallel with cobalt-chromium (CoCr) NPs, and no increase in DNA double-strand breaks was demonstrated in the presence of alumina [38]. Different results were obtained in another study, which showed that at low concentrations (10 μM to 1 mM), the alumina NPs (size and crystallinity unknown) were at the origin of genotoxic effects without decreasing cell viability (except at the highest concentrations) on human peripheral blood lymphocytes [42]. Indeed, these NPs increased single-strand breaks and oxidative DNA damage (2,6-diamino-4-hydroxy-5-N-methylformamidopyrimidine and 7,8-dihydro-8-oxo-2′deoxyguanosine). A third study has demonstrated the induction of DNA strand breaks by Al_2_O_3_ NPs (13 and 50 nm, crystallinities unknown) after incubation with Chinese hamster lung fibroblasts [43]. Moreover, significant oxidative stress (decrease in glutathione, activity of superoxide dismutase, malondialdehyde, and total antioxidant capacity) was demonstrated after exposure to concentrations ranging from 15 to 60 μg/mL. Comparison of cytotoxic and genotoxic effects of four different NPs (oxides of cobalt, iron, silicon, and aluminum) on human lymphocytes in a recent study has, however, demonstrated that Al_2_O_3_ NPs cause less damage to DNA than the other NPs studied [44]. Nevertheless, they do significantly increase the production of reactive oxygen species and lead to a significant decrease in reduced glutathione at a concentration of 100 μg/mL. Negative results were obtained during reverse mutation tests on bacteria in the presence of alumina NPs (sizes less than 50 nm, crystallinity unknown), leading to the conclusion of an absence of mutagenic potential of these NPs [45].

Other specific effects of alumina NPs have been demonstrated in vitro. Thereby, alumina NPs (8–12 nm, crystallinity unknown, concentrations ranging from 1 μM to 10 mM) can induce a decrease in the expression of tight junction proteins [46]. A pre-incubation of HBMEC cells (Human Brain Microvascular Endothelial Cells, brain cell line) with glutathione blocks this effect, meaning that it could be a consequence of a phenomenon related to oxidative stress induced by exposure to NPs. Furthermore, a study carried out on erythrocytes of different species (human, rat, and rabbit) highlighted evidence of a hemolytic power of alumina NPs (13 nm, less than 50 nm, and nanofibers 2–6 nm by 200–400 nm, crystallinities unknown) [47]. Although alumina NPs are metal oxides known for their antimicrobial properties, they have only limited antimicrobial properties [48]. Only high concentrations (1000 μg/mL) have a moderate effect on bacterial proliferation (*Escherichia coli*).

A summary of the results of in vitro studies exploring the cytotoxic effects of alumina particles is available in Table 3.

## 5. Discussion

Biological mechanisms of lung toxicity and physiopathology triggered by exposure to alumina particles are still unclear and not sufficiently studied. The small number of cohort studies complicates the identification of clear exposure–response relationships for respiratory diseases [49]. In human studies, the time of population exposure and associated comorbidities and medical background (asthma, smoker/non-smoker, etc.) are often unknown. These studies mainly addressed worker populations. However, a recent work was carried out by Sikkeland et al. on the sputum of healthy volunteers never-smokers, with no allergy and respiratory diseases, free from respiratory infections 4 weeks prior and with a standardized FVC (Forced Vital Capacity)/FEV 1 ratio of 80 ± 1.9 [9]. To the best of our knowledge, it is to date the only existing study addressing specifically inflammatory effects of Al_2_O_3_ particles (neutrophils and IL-8 increased concentrations) and localized respiratory effects on humans. However, the duration of exposure is only 2 h, while occupational exposure may be for longer durations. Interestingly, they performed sputum collection as increase in neutrophil concentration was characterized among other workers exposed to other pollutants in several different industries, such as paper mills, popcorn factories, cement industry, pig farming, fish feed production, and waste handling. Sputum collection was realized until 24 h after exposure because collecting induced sputum several times within 48 h would be problematic since the sputum induction process may also lead to inflammation [50]. Consequently, sputum collection may be considered to analyze early lung pro-inflammatory effects on humans after exposure, but no further. To analyze the chronic inflammatory response, BALF collection on healthy human volunteers is not considered ethically acceptable as it is invasive and painful. However, such analysis remains essential on anesthetized animals to correctly describe pro-inflammatory mechanisms potentially involved in lung diseases.

Human studies are often not specific to alumina particle toxicity, as workers may inhale a mix of pollutants in the occupational environment. Interestingly, Mazzoli-Rocha et al. exposed by whole-body inhalation BALB/c mice to dust (mainly Al_2_O_3_ particles) collected in an aluminum-producing facility, and they showed impaired lung mechanics associated with inflammation (influx of polymorphonuclear cells) [51]. In order to improve the knowledge of pro-inflammatory effects caused specifically by alumina particle exposure, two studies were performed recently by nose-only inhalation. On the one hand, Kim et al. exposed rats for one month to different concentrations (ranging from 0.2 to 5 mg/m^3^) of Al_2_O_3_ NPs, showing strong inflammatory cytokine secretion in BALF [15]. However, particle crystallinity was not characterized by the authors. Several physico-chemical properties of Al_2_O_3_ NPs are often missing and/or not sufficiently characterized in scientific studies. Particle concentration, size distribution, surface chemistry, and NPs crystallinity seem to have a great impact on biological effects [52]. Alumina has several crystalline phases, and transitions between them occur as follows: γ-Al_2_O_3_ → δ-Al_2_O_3_ → θ-Al_2_O_3_ → α-Al_2_O_3_ [53]. Three other crystal forms also exist but are in the minority: η, χ, and κ [54]. Crystalline phase α-Al_2_O_3_ is the thermodynamically stable one, whereas γ, δ, and θ phases correspond to transition metastable alumina particles [53,55]. Therefore Al_2_O_3_ NPs crystallinity is an important physico-chemical parameter to characterize, as it was shown that these NPs could induce or not fibrotic effects depending on their crystallinities [29]. This was also demonstrated in several human cell lines, including bronchial or alveolar epithelial cells, that cytotoxicity could be modulated depending on the crystallinities of Al_2_O_3_ particles [34,39]. On the other hand, Bourgois et al. exposed rats by nose-only inhalation to a strong concentration of γ/δ-Al_2_O_3_ NPs, also showing increased inflammatory response after five days [14]. However, only one elevated concentration (20 mg/m^3^) of these well-characterized NPs was administrated to animals for only early analysis of pro-inflammatory effects. Different concentrations of Al_2_O_3_ particles could also be administered to rats in order to better establish dose-effects curves and consequently to build regulatory toxicological values. To date, in France, the average exposure limit value is 10 mg/m^3^ for total alumina dusts, which corresponds to the regulatory limit for the metal aluminum. Therefore, animal studies will play a major role in establishing new occupational exposure limit values for alumina particles and nanoparticles.

Pro-inflammatory mechanisms triggered by nose-only inhalation exposure of Al_2_O_3_ NPs seem to involve an increase in neutrophils, lymphocytes, and macrophages afflux in BALF in association with pro-inflammatory cytokines secretion and LDH release [14,15]. This result was also observed after whole-body inhalation of Al_2_O_3_ NPs [16]. Some authors hypothesized that Al_2_O_3_ NPs could stimulate the NFκB pathway [36]. NFκB can contribute to inflammasome regulation, which is involved in IL-1β synthesis [56,57]. This pathway is also known to be activated in the lungs of patients with COPD [58]. The release of IL-1β in BALF may be linked to TNF-α secretion observed in several studies [14,15,28,30]. It has also been demonstrated that in the context of acute inflammation, IL-1β contributes to TNF-α-mediated chemokine release and neutrophil recruitment to the lung [59]. However, IL-6 secretion was not systematically increased in BALF after inhalation of Al_2_O_3_ NPs. We hypothesize that it may be attributed to Al_2_O_3_ NPs concentration administrated to animals, as Li et al. showed increased IL-6 concentration after seven days of exposure to 0.4 mg/m^3^, while no IL-6 increase was found by Bourgois et al. after four days of exposure to roughly 20 mg/m^3^ [14,16]. However, the crystallinity of Al_2_O_3_ NPs used by Li et al. is unknown, and exposure durations are different between both studies. Consequently, the conclusion about the mechanism triggering IL-6 secretion is hard to explain because studies cannot rigorously be compared. Another study was realized on C57Bl/6 J male mice exposed to aluminum oxide-based nanowhiskers (3.3 ± 0.6 mg/m^3^) using a dynamic whole-body exposure chamber for 2 or 4 weeks [60]. These sub-chronic exposures induced an increase in lung macrophage concentration but did not induce an increase in pro-inflammatory cytokines release (i.e., IL-6, IFN-γ, MIP-1α, TNF-α, and MIP-2). This result is contradictory with previous other studies performed on spherical Al_2_O_3_ NPs where pro-inflammatory cytokines (i.e., IL-6, IL-1β, TNF-α, and MIP-2) were released in BALF after one or four weeks of exposure [14,15]. Therefore, the nano-objects shape could also play an important role in Al_2_O_3_ NPs toxicity and associated pro-inflammatory effects on the lungs. Some other cytokines, such as MIP-2 and GRO/KC, may play a role in the early pulmonary inflammation contributing to polymorphonuclear cell recruitment within 24 h after exposure. Several studies demonstrated down-regulation of their secretion in BALF or nasal fluid lavage after several days [14,61]. Overall, in order to study chronic toxic and pro-inflammatory effects of Al_2_O_3_ NPs, it would be interesting to perform longer studies or to house animals longer after inhalation exposure. These studies may allow bettering determining if pro-inflammatory effects are reversible or if diseases such as COPD, emphysema, or pulmonary fibrosis may occur. As alumina is classified in “aluminum production” as carcinogenic to humans (Group 1) by the International Agency for Research on Cancer, long-term studies are essential. To the best of our knowledge, only one long-term study showed that up to one year after intra-tracheal exposure of rats to Al_2_O_3_ particles [29]. None of the five aluminas (α- and γ- crystalline phases) used for primary aluminum production showed any fibrogenic potential, while chemical grade Al_2_O_3_ particles or laboratory-produced samples induced fibrogenic lesions in the lung parenchyma. It would also be interesting to explore after pro-inflammatory and pro-fibrogenic effects of Al_2_O_3_ particles after inhalation that might modify Al_2_O_3_ particles’ lung burden and, consequently, biological effects compared to intra-tracheal instillation exposure. 

Lung pro-inflammatory mechanisms triggered specifically by Al_2_O_3_ particles are not sufficiently explored in in vitro studies. To the best of our knowledge, only Osterling et al. have investigated mRNA and protein expression of adhesion molecules of monocytes on endothelial cells (VCAM-1, ICAM-1, and ELAM-1) [40]. In order to reduce animal experiments and to better understand pro-inflammatory mechanisms and chronic effects, new 3D in vitro models have been recently developed. A recent literature review highlights the benefits of using 3D co-culture models to investigate the complexity of cellular interactions during pulmonary inflammation [62]. A specific in vitro mini-lung fibrosis model equipped with non-invasive real-time monitoring of cell mechanics was developed [63]. This in vitro model combined a co-culture of three cell types: epithelial and endothelial cell lines incubated with primary fibroblasts from idiopathic pulmonary fibrosis patients. Cells are cultivated on a biomimetic ultrathin basement (biphasic elastic thin for air–liquid culture conditions, BETA) membrane (<1 µm) developed with unique properties, including biocompatibility, permeability, and high elasticity (<10 kPa) for cell culturing under air–liquid interface (ALI). This cellular model may allow us to study more precisely pro-inflammatory or pro-fibrogenic mechanisms following exposures to Al_2_O_3_ NPs, taking into account the elasticity of the alveolo-capillary barrier in ALI and real-time measurements. Other studies also suggest using cell co-culture, including fibroblasts, to investigate the inflammatory and pro-fibrogenic effects of inhaled components. For instance, Barosova et al. recently published the development of a three-dimensional alveolar model consisting of human primary alveolar epithelial cells, fibroblasts, and endothelial cells, with or without macrophages [64]. Cell co-cultures are cultivated on bicameral chambers in ALI and mimic the alveolo-capillary barrier. Pulmonary cells can be exposed with the help of specific commercialized devices to particle mist. This type of cellular model could be interesting in exploring long-term cytotoxic and pro-inflammatory mechanisms in vitro.

## 6. Conclusions

This review is a critical update of the current knowledge on underlying toxicological, molecular, and cellular mechanisms induced by exposure to Al_2_O_3_ NPs on the lungs. Human and animal studies point out that inhalation of Al_2_O_3_ particles can induce aluminosis, local respiratory symptoms (cough, shortness of breath), and pro-inflammatory response and may trigger long-term pulmonary fibrosis. Not enough cohort studies and clinical studies on healthy volunteers are performed to better understand these mechanisms and to establish clear exposure–response relationships. In studies with animals or cells, physico-chemistry of Al_2_O_3_ particles has to be extensively analyzed and published in order to improve the understanding of related biological effects. Inhalation exposures are closer to realistic environmental exposures, and long-term animal studies are necessary to determine whether pro-inflammatory reactions may reverse or turn into fatal diseases such as pulmonary fibrosis. Three-dimensional co-culture models may also allow studying these underlying pro-inflammatory and cytotoxic mechanisms for several weeks of exposure, as it was recently performed [64,65]. Consequently, in order to improve the analysis of pro-inflammatory and pro-fibrogenic effects, a combination of long-term animal studies by inhalation exposures and the use of dedicated 3D co-culture models is needed. 

## Figures and Tables

**Table 1:** Summary of clinical studies on health effects after inhalation of alumina particles.

References	Studied Atmospheres	Population (Size)	Health/Biological Effects	Particle Size	Particle Concentration	Exposure Time
Sikkeland et al., 2016 [9]	Al_2_O_3_ particles	Healthy volunteers (15)	Increases in neutrophils, total proteins, and IL-8 concentrations in the sputum. Localized respiratory effects, no systemic effect.	3.2 μm	3.9 mg/m^3^	2 h
Jederlinic et al., 1990 [17]	Complex aerosols containing Al_2_O_3_ particles	Workers (9)	Prevalence of pulmonary fibrosis 300 times higher than that observed in the general population	Unknown	Unknown	25 years
Hull et al., 2002 [3]	Aluminum welding fumes	Workers (2)	Pneumoconiosis cases	10 nm–1 μm (Al, aggregates)	Unknown	22–24 years
Vallyathan et al., 1982 [11]	Aluminum welding fumes	Worker (1)	Pulmonary fibrosis case	Unknown	Unknown	Unknown
Townsend et al., 1985 [18]	Aluminum welding fumes (containing mainly Al_2_O_3_ particles)	Workers (1142)	Higher frequency of respiratory diseases, such as chronic obstructive pulmonary disease (COPD). Decrease in lung function.	Unknown	Unknown	Unknown
Schwarz et al., 1998 [19]	Complex aerosols containing Al_2_O_3_ particles and hard metals	Workers (14)	Diffuse pulmonary interstitial inflammatory changes in five workers (2 asymptomatic, 1 symptomatic with giant cell inflammation, and 2 with local inflammation).	Unknown	Unknown	Unknown
Hunter et al., 1944 [8]	Complex aerosols containing aluminum and alumina NPs	Workers (50)	Respiratory symptoms (cough, shortness of breath). No proven correlation with appearance of respiratory diseases.	0.23–0.5 μm (Total particles)	400–2430 /cm^3^ (Total particles)	6–39 years
Edling et al., 1987 [20]	Complex aerosols containing alumina particles, silicon carbides, and formaldehyde	Workers (521)	No increase in total or cancer mortality nor incidence of non-malignant respiratory disease.	Unknown	1 mg/m^3^ (Total particles)	25 years
Sjorgen et al., 1985 [21]	Aluminum welding fumes	Workers (259)	Respiratory symptoms with no alteration of lung function.	<1 μm (Total particles)	0–42 mg/m^3^ (Total particles)	1–41 years

**Table 2:** Summary of animal studies.

References	Exposure Method	Animal Model	Biological Effects	Primary Particle Size	Particle Concentration	Particle Crystallinity	Exposure Time
Kim et al., 2018 [15]	Nose-only inhalation	Rats	Pro-inflammatory effects. Increases in neutrophils, lymphocytes, LDH, TNF-α, and IL-6 in BALF. Histopathological lesions with alveolar macrophage accumulation.	11.94 nm	0.2–5 mg/m^3^	Unknown	28 days (5 days/week)
Bourgois et al., 2021 [14]	Nose-only inhalation	Rats	Pro-inflammatory effects. Increases in neutrophils, macrophages, IL-1β, TNF-α, GRO/KC, and MIP-2 in BALF. Histopathological lesions with neutrophil accumulation at the interstitial and alveolar level, as well as by a thickening of alveolar partitions.	13 nm	20–22.1 mg/m^3^	γ/δ	4 h; 4 h/day during 4 days
Li et al., 2017 [16]	Whole-body inhalation	Mice	Emphysema, small airway remodeling, enhanced inflammation, and apoptosis. PTN6 down-regulation, STAT3 phosphorylation, and PDCD4 apoptotic marker increased expression. Increases in IL-6 and IL-33 concentrations in BALF. COPD-like lesions.	40 nm	0.4 mg/m^3^	Unknown	7 days
Tornling et al., 1993 [27]	Intra-tracheal instillation	Rats	Increases in neutrophils, macrophages, and fibronectin concentrations in BALF. Persistence of this phenomenon 12 months after intra-tracheal instillation.	4.37 μm	40 mg	γ/α	_
Cho et al., 2012 [7]	Intra-tracheal instillation	Rats	Increase in neutrophil concentration. Hemolytic potential of alumina NPs.	6.3 nm	150 cm^2^ (0.5 mL at 300 cm^2^/mL)	Unknown	_
Kwon et al., 2013 [28]	Nasal instillation	Rats	Increase in total proteins, LDH, IL-6, and TNF-α concentrations in BALF.	Unknown	1–40 mg/kg	Unknown	_
Ess et al., 1993 [29]	Intra-tracheal instillation	Rats	Particles usually used for the manufacture of aluminum (α and γ) had no fibrotic effect, whereas other particles tested could induce this type of lesion. No link between cytotoxicity and fibrotic effect.	<11 μm	50 mg/0.5 mL 1% suspension	α, γ, δ, and χ	_
El-Hussainy et al., 2016 [30]	Intra-tracheal instillation	Rats	ECG disorders and an increase in myocardial (LDH, triglycerides, creatine phosphokinase, cholesterol, nitric oxide) and inflammatory (TNF-α) damage markers in blood serums. Decrease in antioxidants (reduced glutathione and superoxide dismutase) in animal serum.	11 nm	30 mg/kg/day	α	14 days

**Table 3:** Summary of in vitro studies.

References	Cell Model	Biological Effects	Primary Particle Size	Particle Concentration	Particle Crystallinity	Exposure Time
Radziun et al., 2011 [31]	Murine fibroblasts (L929 cell line) and normal human skin fibroblasts (BJ cells)	No decrease in cell viability or apoptosis induction. NPs internalization in both cell types.	50–80 nm	10–400 µg/mL	γ	24 h
Bohme et al., 2014 [32]	Human alveolar epithelial cells (A549 cell line) and human skin keratinocytes (HaCaT cell line)	Internalization in cell cytoplasm, no detection in cell nuclei, no cytotoxicity.	14 nm 111 nm 750 nm	10–50 mg/L	α and α/δ	24 h
Kim et al., 2010 [33]	Human lung cell lines (A549 carcinoma cells and L-132 normal cells)	Lower cytotoxicity of NPs of alumina compared to the other metal oxide NPs tested (CeO_2_, TiO_2,_ and ZnO).	20 nm	0.5–1000 µg/mL	Unknown	24 h, 48 h, and 72 h
Park et al., 2016 [34]	Six human cell lines, including bronchial epithelial BEAS-2B cells	γ-aluminum oxide hydroxide nanoparticles induced greatest toxicity compared to γ- and α- Al_2_O_3_ NPs.	180–200 nm	5 and 20 µg/mL	α and γ	24 h
Otero-Gonzalez et al., 2012 [35]	Human bronchial epithelial cells (169HBE14o- cell line)	Decrease in normalized cell index and cell viability at the highest concentrations.	<50 nm	100–1000 mg/mL	Unknown	48 h
Simon-Vazquez et al., 2016 [36]	Human pleural cells (NCI-H460 cell line)	No modification of normalized cell index.	14 nm	15, 63, and 500 µg/mL	Unknown	48 h
Wei et al., 2014 [37]	Human alveolar epithelial cells (A549 cell line)	Smallest NPs more cytotoxic (inhibition of cell proliferation). Hydrodynamic diameter does not influence cytotoxicity. Al_2_O_3_ NPs more cytotoxic compared to TiO_2_ NPs.	10 and 50 nm	0.1–10 mg/mL	γ and γ/δ	2 and 5 days
Tsaousi et al., 2010 [38]	Primary human fibroblasts	No induction of micronuclei and no increase in DNA double-strand breaks. Size and shape of Al_2_O_3_ nano-objects do not influence genotoxicity (micronuclei and chromosomal aberration).	0.2 nm and 2 µm and alumina fibers (0.9 µm diameter, 12.03 µm length)	1.33–133.33 µg/cm^2^	Unknown	24 h
Oesterling et al., 2008 [40]	Primary pulmonary artery endothelial cells, human umbilical vein endothelial cells, and monocytes	Increases in mRNA and protein expression of VCAM-1, ICAM-1, and ELAM-1 increased adhesion of activated monocytes.	10–20 nm	1–250 µg/mL	α/γ	24 h
Yun et al., 2020 [41]	Human bronchial epithelial cells	Up-regulation of homologous miRNA in *Homo sapiens* and *Mus musculus* miR-297.	5–100 nm (scanning electron microscopy)	50 and 250 μg/mL	Unknown	24 h
Sliwinska et al., 2015 [42]	Human peripheral blood lymphocytes	Increased single-strand breaks and oxidative DNA damage (2,6-diamino-4-hydroxy-5-N-methyl formamidopyrimidine and 7,8-dihydro-8-oxo-2′deoxyguanosine).	Unknown	From 10 μM to 1 mM	Unknown	24 h
Zhang et al., 2017 [43]	Chinese hamster lung fibroblasts, *Salmonella typhimurium*	Genotoxicity of Al_2_O_3_ NPs (Ames test, Comet test, Micronucleus assay, Sperm deformity test). Antioxidant decreases.	13 nm; 50 nm	0.5–5000 μg/mL	Unknown	12 h, 24 h, and 48 h
Rajiv et al., 2016 [44]	Human lymphocytes	Al_2_O_3_ NPs cause less damage to DNA than the other NPs studied (Co_3_O_4_, Fe_2_O_3,_ and SiO_2_ NPs). Al_2_O_3_ NPs exposures induced significant increases in reactive oxygen species production.	<50 nm	10–100 μg/mL	Unknown	24 h
Pan et al., 2010 [45]	*Salmonella typhimurium*	Negative reverse mutation assay: absence of mutagenic potential.	<50 nm	10–1000 μg/plate	Unknown	72 h
Chen et al., 2008 [46]	Human Brain Microvascular Endothelial Cells (HBMEC cell line)	Decrease in the expression of tight junction proteins related to oxidative stress induced.	8–12 nm	From 1 μM to 10 mM	Unknown	24 h
Vinardell et al., 2015 [47]	Erythrocytes (Human, Rat, Rabbit)	Hemolytic power of Al_2_O_3_ NPs.	13 nm; <50 nm; Nanofibers (2–6 nm diameter, 200–400 nm length)	2.5–40 mg/mL	Unknown	1 h, 3 h, and 24 h
Sadiq et al., 2009 [48]	*Escherichia coli*	Weak antimicrobial power at high concentration.	<50 nm	10–1000 μg/mL	γ	24 h
Bourgois et al., 2019 [39]	Human alveolar epithelial cells (A549 cell line)	No effect on cell index, cell viability, reduced glutathione, and double DNA strand breaks. Internalization of NPs in cytoplasm.	10 nm; 13 nm; 500 nm	1.56–200 µg/cm^2^	γ and γ/δ	24 h
